# Methylation determines the extracellular calcium sensitivity of the leak channel NALCN in hippocampal dentate granule cells

**DOI:** 10.1038/s12276-019-0325-0

**Published:** 2019-10-10

**Authors:** Seul-Yi Lee, Tuan Anh Vuong, Xianlan Wen, Hyeon-Ju Jeong, Hyun-Kyung So, Ilmin Kwon, Jong-Sun Kang, Hana Cho

**Affiliations:** 1 Department of Physiology, Sungkyunkwan University, Suwon, Korea; 2 Single Cell Network Research Center, Sungkyunkwan University, Suwon, Korea; 3 Department of Molecular Cell Biology, Sungkyunkwan University, Suwon, Korea; 4 Department of Anatomy and Cell Biology, Sungkyunkwan University, Suwon, Korea

**Keywords:** Ion channels in the nervous system, Methylation

## Abstract

The sodium leak channel NALCN is a key player in establishing the resting membrane potential (RMP) in neurons and transduces changes in extracellular Ca^2+^ concentration ([Ca^2+^]_e_) into increased neuronal excitability as the downstream effector of calcium-sensing receptor (CaSR). Gain-of-function mutations in the human NALCN gene cause encephalopathy and severe intellectual disability. Thus, understanding the regulatory mechanisms of NALCN is important for both basic and translational research. This study reveals a novel mechanism for NALCN regulation by arginine methylation. Hippocampal dentate granule cells in protein arginine methyltransferase 7 (PRMT7)-deficient mice display a depolarization of the RMP, decreased threshold currents, and increased excitability compared to wild-type neurons. Electrophysiological studies combined with molecular analysis indicate that enhanced NALCN activities contribute to hyperexcitability in PRMT7−/− neurons. PRMT7 depletion in HEK293T cells increases NALCN activity by shifting the dose-response curve of NALCN inhibition by [Ca^2+^]_e_ without affecting NALCN protein levels. In vitro methylation studies show that PRMT7 methylates a highly conserved Arg1653 of the NALCN gene located in the carboxy-terminal region that is implicated in CaSR-mediated regulation. A kinase-specific phosphorylation site prediction program shows that the adjacent Ser1652 is a potential phosphorylation site. Consistently, our data from site-specific mutants and PKC inhibitors suggest that Arg1653 methylation might modulate Ser1652 phosphorylation mediated by CaSR/PKC-delta, leading to [Ca^2+^]_e_-mediated NALCN suppression. Collectively, these data suggest that PRMT7 deficiency decreases NALCN methylation at Arg1653, which, in turn, decreases CaSR/PKC-mediated Ser1652 phosphorylation, lifting NALCN inhibition, thereby enhancing neuronal excitability. Thus, PRMT7-mediated NALCN inhibition provides a potential target for the development of therapeutic tools for neurological diseases.

## Neuronal function: Keeping ion channel activity in check

The addition of a methyl group to an arginine residue on the ion channel NALCN contributes to suppress the activity of this membrane protein and reduces neuronal excitability. Hana Cho, Jong-Sun Kang and colleagues at Sungkyunkwan University in South Korea found that neurons in the hippocampus of mice lacking an enzyme that mediates the transfer of methyl groups to proteins have increased NALCN activity and are more likely to fire an electrical signal. Furthermore, they showed that NALCN methylation facilitates the phosphorylation of an adjacent amino acid that prevents channel activation in response to extracellular calcium concentrations. These findings suggest that NALCN methylation has a key role in regulating the channel’s sensitivity to calcium. Moreover, they reveal a new mechanism for regulating neuronal excitability that could be targeted therapeutically to ameliorate diseases characterised by neuronal hyperexcitability.

## Introduction

NALCN is a nonselective sodium leak channel that is highly conserved evolutionarily and is expressed widely in neurons throughout the brain^1^. NALCN acts as a downstream effector of the calcium-sensing receptor (CaSR). CaSR detects [Ca^2+^]_e_ and regulates NALCN activities in response to small changes in [Ca^2+^]_e_ levels. NALCN, in turn, depolarizes the resting membrane potential (RMP) and increases neuronal excitability. There is growing evidence that NALCN is involved in many processes, such as locomotor behaviors, sensitivity to volatile anesthetics, and respiratory rhythms^2^. There is also evidence that alterations in NALCN activity can cause a wide variety of diseases. For example, gain-of-function mutations in the human NALCN gene cause encephalopathy and severe intellectual disability^3,4^. Thus, understanding the regulatory mechanisms of NALCN activity is important for both basic and translational research. NALCN channel activities are potentiated by substance P^5^, while they are inhibited by the dopamine (D2) receptor^6^. However, the regulatory mechanisms of NALCN activity remain mostly unresolved.

Diverse mechanisms have been linked to the control of ion channel activity. One mechanism is related to the control of expression and membrane targeting, leading to alterations in ion-channel density at the membrane^7^. In addition, the posttranslational modification of ion channels has been shown to play important roles in the control of a channel’s functional properties^8–10^. A number of modifications, including phosphorylation, ubiquitylation and/or sumoylation, have been identified as modulating ion channel conductance^11,12^. Arginine methylation has newly emerged as a key posttranslational modification that can regulate ion channel activity. Protein arginine methylransferases (PRMTs) are enzymes that catalyze the mono- and di-methylation of arginine residues of histone or nonhistone substrates^13^. Among the nine characterized members, PRMTs can be classified into three types based on their catalytic activity. Type I PRMTs (PRMT1, PRMT2, PRMT3, PRMT4, PRMT6, and PRMT8) catalyze the formation of asymmetrically di-methylated arginine residues on substrate proteins, and type II PRMTs (PRMT5 and PRMT9) catalyzes symmetrically di-methylated arginine residues. Finally, the type III PRMT, including PRMT7, catalyzes the mono- and symmetrically di-methylated arginine residues. Recently, we and others demonstrated the physiological roles for PRMT1, PRMT5 and PRMT8 in the control of neural function and neurodegenerative diseases^14–16^. Lin et al. and Bowitch et al. showed critical roles of PRMT8 and PRMT5 in the neural development of zebrafish^15^ and of SER-2 tyramine receptor-mediated behaviors in *Caenorhabditis elegans*^16^, respectively. We have demonstrated that the PRMT1-mediated arginine methylation of the KCNQ channel contributes to the stabilization of neuronal excitability^14^. Thus, PRMTs are potentially intriguing targets for the modulation of channel activity and neural function.

The potential involvement of PRMT7 in neural function has been proposed by a recent study reporting a male with severe intellectual disability, facial dysmorphism, microcephaly, short stature, brachydactyly, cryptorchidism and seizures who was found to have a homozygous deletion of 15,309 base pairs encompassing the transcription start site of PRMT7, resulting in functionally a null allele^17^. Thus, in this study, we examined the role of PRMT7 in neuronal excitability in hippocampal dentate gyrus (DG) granule cells using mice null for PRMT7. Our results demonstrate that PRMT7−/− (KO) DG neurons exhibit increased intrinsic excitability compared to wild type (WT) DG neurons. A molecular mechanism study revealed that PRMT7 is critical for the control of the Na^+^ leak channel, NALCN. PRMT7 binds to NALCN and methylates the arginine residue 1653 in its cytoplasmic tail, which is critical for the regulation of NALCN’s [Ca^2+^]_e_ sensitivity. Taken together, our study demonstrates that the methylation of arginine on NALCN decreases the magnitude of the depolarizing current for a given extracellular calcium molecule, leading to a membrane-stabilizing effect.

## Materials and methods

### Animal studies

Prmt7^<tm1a(EUCOMM)Wtsi>^ mice were purchased from Sanger Institute (Wellcome Trust Sanger Institute Hinxton, Cambridge, UK). From heterozygous crosses, wild-type or heterozygous littermates were used as controls for the phenotype studies of Prmt7 knockout mice. All animal experiments were approved by the Institutional Animal Care and Use Committee (IACUC) of Sungkyunkwan University School of Medicine (SUSM).

### Brain slice preparation and recording

Brain slices were prepared from male KO and WT mouse littermates 5–8 weeks of age. Mice were sacrificed by decapitation after being anesthetized with pentobarbital sodium, and the whole brain was immediately removed from the skull and chilled in artificial cerebrospinal fluid (aCSF) at 4 ˚C. Transverse hippocampal slices (350 μm thick) were prepared using a vibratome (VT1200S, Leica, Germany, Nussloch). Slices were incubated at 35 ˚C for 30 min and were thereafter maintained at 32 ˚C until in situ slice patch recordings and fluorescence microscopy were performed. Hippocampal granule cells from the DG were visualized using an upright microscope equipped with differential interference contrast optics (BX51WI, Olympus, Japan, Tokyo). Whole-cell current clamp techniques were used to measure the excitability of dentate granule cells. The pipette solution contained (in mM): 143 K-gluconate, 7 KCl, 15 HEPES, 4 MgATP, 0.3 NaGTP, 4 Na-ascorbate, and 0.1 EGTA/or 10 BAPTA with the pH adjusted to 7.3 with KOH. The bath solution (or aCSF) for the control experiments contained the following (in mM): 125 NaCl, 25 NaHCO_3_, 2.5 KCl, 1.25 NaH2PO4, 2 CaCl_2_, 1 MgCl_2_, 20 glucose, 1.2 pyruvate, and 0.4 Na-ascorbate, pH 7.4 when saturated with carbogen (95% O_2_ and 5% CO_2_). All bath solutions included 20 μM bicuculline and 10 μM CNQX to block inhibitory synaptic signals. The perfusion rate of the bathing solution and the volume of the recording chamber for slices were 2.2 ml/min and 1.2 ml, respectively. Patch pipettes with a tip resistance of 3–4 MΩ were used. The series resistance (Rs) after establishing the whole-cell configuration was between 10 and 15 MΩ. Electrophysiological recordings were made in somata with an EPC-8 amplifier (HEKA Instruments, Lambrecht/Pfalz, Germany). Experiments were performed at 32 ± 1 °C. The following parameters were measured: (1) the resting membrane potential, (2) AP threshold current (current threshold for single AP generation, 100 ms duration), (3) the input resistance (R_in_, membrane potential changes (V) for a given hyperpolarizing current (35 pA, 600 ms) input), (4) AP threshold potential, (5) AP height, defined as the peak relative to the most negative voltage reached during the afterhyperpolarization immediately after the spike, (6) AP half-width, measured as the width at half-maximal spike amplitude, and (7) F-I curve (firing frequencies (F) against the amplitude of injected currents (I); 100–250 pA). We excluded data for analysis when the series resistance exceeded 20 MΩ or when the RMP was more positive than 60 mV. The whole-cell voltage clamp technique was used to measure K^+^ currents. Whole-cell K^+^ currents, evoked in response to voltage steps to potentials ranging from –70 mV to +30 mV (in 10 mV increments, 1 s duration) from a holding potential of –70 mV, were examined in WT and KO granule cells. Tris-HCl was used to replace 125 mM NaCl in the baths containing a 5 mM Na^+^ bath. Similar results were obtained when N-methyl-D-glucamine (NMDG) was used to replace Na^+^. TTX (1 μM) and ABC mix (10 μM APV, 20 μM bicuculline, 20 μM CNQX) were applied in the bath to block Na_v_ and synaptic currents. The I_L-Na_ leak current was measured by subtracting the currents recorded in low (0 mM) [Na^+^]_e_ from those in high (125 mM) [Na^+^]_e_ at holding potentials (ΔI_L-Na_)^18^. The low [Ca^2+^]_e_-activated current, I_LCA_, was measured as the change in the size of ΔI_L-Na_ or the change in the holding current (in bath containing 125 mM Na^+^) when [Ca^2+^]_e_ was lowered as indicated.

### Generation of NALCN mutants, cell culture, transfection, and recording

To construct various point mutants for NALCN, the pCMV2-NALCN expression vector was used as the template using the QuikChange Site-Directed Mutagenesis Kit (Agilen) as previously described^19^. The primers used are listed in Supplementary Table 1.

HEK293T cells (ATCC, USA, Virginia) were cultured as previously described^14^. Cells were maintained in Dulbecco’s modification of Eagle’s medium (Invitrogen, USA, California) supplemented with 10% fetal bovine serum. Lentiviruses of shRNA control and PRMT7 (shPRMT7) were generated with a modified lentiviral vector derived from pLKO.1 (Sigma-Aldrich, St. Louis, MO) in HEK293T cells using the helper plasmids pCMV-VSVG and pCMV delta 8.2 using Lipofectamine 2000 reagents (Invitrogen, USA, California). To generate stable cell lines, lentivirus particles were mixed in medium in the presence of 8 μg/ml polybrene (Sigma-Aldrich, St. Louis, MO) for 2 days and then selected in 2 μg/ml puromycin-containing medium.

For NALCN recording, NALCN, UNC80, a constitutively active Src kinase (Src529, bearing a Y529F mutation), and CaSR cDNA were transiently cotransfected using Lipofectamine 2000 reagents as previously described^20^. The NALCN and CaSR constructs were constructed with a vector based on pTracer-CMV2 (Invitrogen) modified to express eGFP (for NALCN) or mCherry RFP (for CaSR) under separate promoters. To obtain a heterologous KCNQ2/3 configuration, human KCNQ2 and KCNQ3 subunits with GFP plasmid were cotransfected in 293T cells and were recorded within 48–72 h after transfection as previously described^21^. The NALCN and KCNQ currents from HEK293T cells were measured with the whole-cell patch clamp technique. Voltage clamp was performed using an EPC-10 amplifier (HEKA Instruments, Germany, Lambrecht/Pfalz) at a sampling rate of 10 kHz filtered at 1 kHz. Data were acquired using an IBM-compatible computer running Patchmaster software (HEKA Instruments, Germany, Lambrecht/Pfalz). The patch pipettes were pulled from borosilicate capillaries (HilgenbergGmbH, Germany, Malsfeld) using a Narishige puller (PC-10, Narishige, Japan, Tokyo). The patch pipettes had a resistance of 2–3 MW when filled with the pipette solution containing (in mM) 140 KMeSO4, 20 KCl, 20 HEPES, 0.5 Na-GTP, 5 Mg-ATP, 4 vitamin C, and 10 1,2-bis (2aminophenoxy) ethane N,N,N_,N_-tetraacetic acid (BAPTA), pH 7.4 adjusted with KOH. The normal external solution was as follows (in mM): 143 NaCl, 5.4 KCl, 5 HEPES, 0.5 NaH2PO4, 11.1 glucose, 0.5 MgCl_2_, and 1.8 CaCl_2_, pH 7.4 adjusted with NaOH. Pipette capacitance was compensated after formation of a gigaohm seal. Access resistance was typically 2.8–3.2 MΩ. The perfusion system was a homemade 100-ml perfusion chamber through which the solution flowed continuously at 5 ml/min. The currents from HEK293T cells were studied by holding the cell at 60 mV, and 1-s steps from 70 to 40 mV in 10-mV increments were applied, followed by 1-s pulses to 60 mV. All recordings were carried out at room temperature (RT). Currents were analyzed and fitted using Patch master (HEKA Instruments, Germany, Lambrecht/ Pfalz) and Origin (ver. 6.0, Microcal, USA, Massachusetts) software. All values are given as the mean ± standard error. The I/V relationship was obtained by plotting the outward current at the end of a 1-s test pulse as a function of the test potential.

### Immunofluorescence staining, immunoprecipitation, surface biotinylation, and immunoblotting

Immunostaining was performed as previously described^22^. Briefly, mouse brains were fixed with 4% paraformaldehyde and dehydrated through an ascending sucrose series followed by cryo-embedding and sectioning with 10 µm thickness on a cryostat microtome (Leica). Sections were permeabilized and processed for incubation with primary antibodies and secondary antibodies (Invitrogen). The antibodies used in this study are listed in Supplementary Table 2. Confocal images were captured under a Zeiss LSM-710 Meta confocal microscope.

Immunoblot analysis was performed as previously described^23^. Briefly, cells or brain tissues were lysed in RIPA buffer (iNtRON Biotechnology, Korea) containing complete protease inhibitor cocktail (Roche), followed by SDS-PAGE and incubated with primary and secondary antibodies. The primary antibodies used in this study are listed in Supplementary Table 2. Immunoprecipitation was performed as described^24^. Briefly, cell lysates were immunoprecipitated with 1μg primary antibody or control IgG at 4 °C overnight, followed by incubation with protein A/G agarose beads (Roche). The precipitates were washed and analyzed by immunoblotting.

For the surface biotinylation assay, HEK293T cells were incubated with 1 mg/ml NHS-LC-biotin (Thermo) in PBS supplemented with 1 mM CaCl_2_ and MgCl_2_ at 4 °C for 30 min^25^. After quenching three times with 100 mM glycine, cells were lysed in lysis buffer, and cell lysates were incubated with streptavidin-agarose beads (Pierce) at 4 °C for 1 h. Beads were washed three times with lysis buffer and analyzed by SDS-PAGE and immunoblotting.

### Protein expression, purification, and in vitro methylation assay

Protein expression, purification and in vitro methylation assays were performed as previously described^14,26^. Briefly, the C-terminal region of rat-NALCN encompassing residues 1588 to 1713 was cloned into the pET-GST plasmid, transformed into *Escherichia coli* (*E. coli*) strain BL21 (DE3, Invitrogen, California, USA), and grown and induced with 0.5 mM isopropyl β-D-1-thiogalactopyranoside (IPTG) at 18 °C for 24 h. Bacterial cell pellets were resuspended in buffer A (50 mM Tris-HCl pH 8.0, 150 mM NaCl, 1 mM EDTA, and 1 mM PMSF) followed by sonication with 60% amplitude for 5 min on ice, and GST-tagged NALCN proteins were purified by binding to 1 ml GSTrap^TM^ FF (GE Healthcare, Uppsala, Sweden), washing with buffer A and eluting with buffer B (50 mM Tris-HCl pH 8.0, 150 mM NaCl, 20 mM Glutathione). Finally, proteins were desalted through a PD-10 column (GE Healthcare) with buffer C (25 mM Tris-HCl pH 8.0, 100 mM NaCl).

An in vitro methylation assay was performed as previously described^26^. Briefly, GST-PRMT7 (10 μg, 2.5 μM final concentration), total histone (10 μg), GST (5.7 μg, 5.1 μM final concentration), GTS-NALCN WT or MT (8.1 μg, 5.6 μM final concentration) and 2 μl [^3^H] AdoMet in 40 μl reaction buffer (50 mM potassium HEPES pH 7.5, 10 nM NaCl, 1 mM DTT). The reaction was incubated at 23 °C for 20–22 h, quenched by the addition of 10 μl of 5X Laemmli sample buffer, boiled and separated on polyacrylamide gel. The gels were then stained with Coomassie Blue. The destained gels were treated in autoradiography-enhancing buffer (EN3HANCE, PerkinElmer) for 1 h and vacuum-dried on 3 MM papers (Whatman, GE Healthcare) at 80 °C for 1 h. The dried gels were then exposed to X-ray film (AGFA Healthcare) to detect methylated protein by autoradiography at −80 °C for 30 days.

### Statistical analysis

All data analysis and curve fittings were performed using Origin 6.0 and Igor pro. The results were expressed as the means ± SEM, two-tailed Student’s *t*-test was used for statistical significance, and the *P* values are given in the figure legends.

## Results

### Elevated intrinsic excitability in KO DG granule cells

**Fig. 1:**
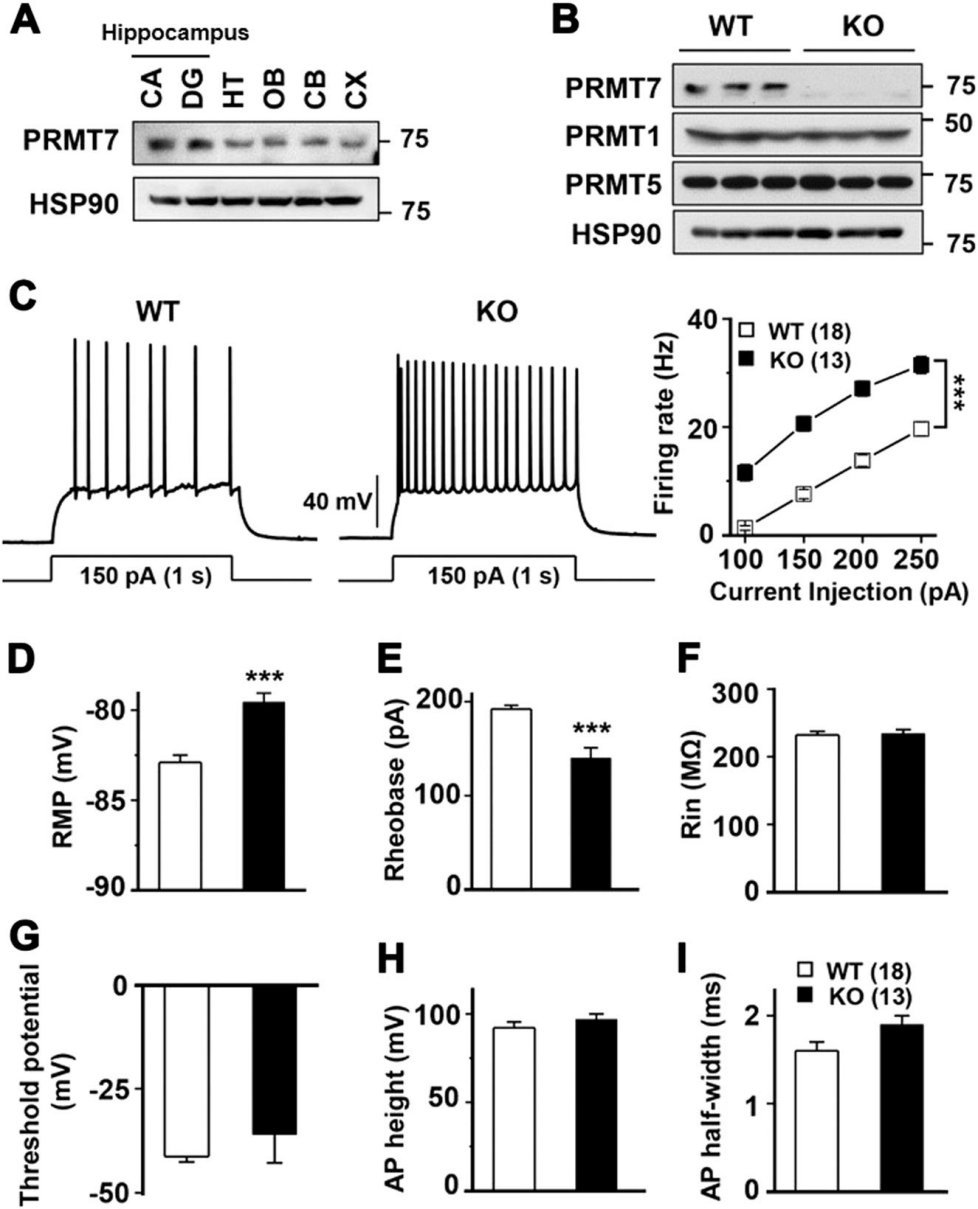
Increased firing frequency in PRMT7 KO dentate granule cells. **a** Immunoblot analysis for PRMT7 expression in 2-month-old mouse brains. CA cornu ammonis, DG dentate gyrus, HT hypothalamus, OB olfactory bulb, CB cerebellum, CX cerebral cortex. **b** Immunoblot analysis for PRMT7, PRMT1, and PRMT5 in PRMT7^−/−^ (KO) DG compared to PRMT7^+/+^ (WT) control mice. HSP90 serves as a loading control. **c** Representative trace in the whole-cell current-clamp recording in mature WT and KO dentate granule cells in response to 1-s depolarizing current injection (150 pA). (Right) The mean number of action potentials (AP No.) plotted against the eliciting currents (from 100 pA to 250 pA, +50 pA increment, during 1-s). At all amplitudes, the mean ± S.E.M. AP No. is significantly higher in KO (■; *n* = 13) than WT granule cells (□; *n* = 18). **d**–**i** The mean value of resting membrane potential (**d**), threshold current for AP generation (100 ms duration; **e**), input resistance (**f**), threshold potential (**g**), AP height (**h**), and AP half-width (**i**) in mature WT and KO granule cells. ***p < 0.001

We first examined the expression of PRMT7 in various brain areas isolated from adult mice. Immunoblot analysis demonstrated that PRMT7 proteins were expressed in all examined brain areas (the CA and DG of the hippocampus, hypothalamus, olfactory bulbs, cerebellum, and cortex); however, PRMT7 is highly expressed in the hippocampus and cortex. (Fig. 1a). As expected, KO brains expressed diminished levels of PRMT7 proteins without alterations in PRMT1 and PRMT5 protein levels (Fig. 1b). Next, we assessed the effect of PRMT7 deletion on neuronal activity using electrophysiological recordings of mature DG granule cells in hippocampal slices. Granule cells from WT mice typically displayed tonic firing patterns in response to a 1-s square current pulse injection: action potential (AP) frequency increased as the magnitude of the square pulse increased (Fig. 1c). KO granule cells showed significantly higher AP frequencies than those from WT mice. To distinguish mature granule cells from young granule cells, we used an input resistance (Rin) of less than 300 MΩ as a criterion for mature granule cells^27^. The average AP frequency in response to a 150 pA depolarizing current in WT granule cells was 7.1 ± 0.8 Hz (*n* = 18), while it significantly increased to 19.3 ± 1.3 Hz (*n* = 13, *p* < 0.001) in KO granule cells. These data indicate that PRMT7 depletion led to enhanced intrinsic excitability in hippocampal DG neurons. The increased firing frequency was accompanied by a depolarization of the RMP (Fig. 1d) and a reduction in the threshold current for the generation of a single AP (rheobase) (Fig. 1e). Rin, threshold potential, and AP shapes, as measured by overshoot and AP half-width, were not affected in KO granule cells (Fig. 1f–i). These results suggest that ion channels active at subthreshold voltages, but not those involved in APs, were altered in the KO granule cells. Furthermore, the pharmacological inhibition of PRMT7 with 100 μM DS437, an inhibitor of PRMT5 and PRMT7 in WT granule cells, caused a similar effect on neuronal excitability and passive electrical properties (SI Appendix, Fig. S1), confirming that the reduction in PRMT7 activity results in enhanced excitability in hippocampal neurons.

### Unlike PRMT1, PRMT7 is not involved in the regulation of KCNQ currents

We have previously demonstrated that granule cells of PRMT1+/− mice showed neuronal hyperexcitability due to altered KCNQ channel regulation^14^. Thus, we examined whether PRMT7 deficiency caused neuronal hyperexcitability through the dysregulation of neuronal KCNQ2/KCNQ3 channels. To do so, HEK293T cells expressing the KCNQ2/3 channel were treated with DS437 or furamidine, a PRMT1-specific inhibitor, and using the conventional whole-cell patch clamp technique, the channel function was measured by applying ‘step’ pulses from –70 to +40 mV in 10-mV increments at a holding potential of –60 mV for 1 s, followed by a tail pulse to –60 mV. Supplementary Fig. 2 demonstrates representative whole-cell current traces recorded from KCNQ2/3-transfected HEK293T cells. Consistent with the previous study^14^, KCNQ2/3-transfected cells displayed slowly activating outward currents and tail currents (SI Appendix, Fig. S2A). Treatment with DS437 did not affect KCNQ2/3 currents that were confirmed by 10 μM XE991 treatment. Assuming that the XE991-sensitive portion entirely represents KCNQ2/3 currents, DS437 inhibited KCNQ2/3 currents by −5.5 ± 1.8% (*n* = 6), and this inhibition was significantly smaller than the inhibition induced by furamidine treatment (72.1 ± 2.1% (*n* = 8), *p* < 0.01) (SI Appendix, Fig. S2B). In addition, the effect of KCNQ inhibition on the neuronal excitability of granule cells from WT and KO mice was examined. Consistent with previous reports (11, 51), the application of XE991 significantly increased the firing frequency of WT neurons (SI Appendix, Fig. S2C and D). For example, the AP frequency in response to a 150 pA-depolarizing current was 2.4 ± 1.2 Hz in the controls and increased to 5.5 ± 1.6 Hz following XE991 treatment. This XE991-induced increase in AP frequency was also observed in the mutant neurons (SI Appendix, Fig. S2E and F); before and after XE991 treatment, the AP frequency in response to a 150 pA depolarizing current was 18.8 ± 2.5 Hz and 23.7 ± 2.2 Hz, respectively. These results demonstrate that KCNQ channel activities are not altered in KO granule cells, suggesting that other ion channels might contribute to the neuronal hyperexcitability observed in KO granule cells.

### Na^+^ leak channel activities are increased in KO granule cells

**Fig. 2:**
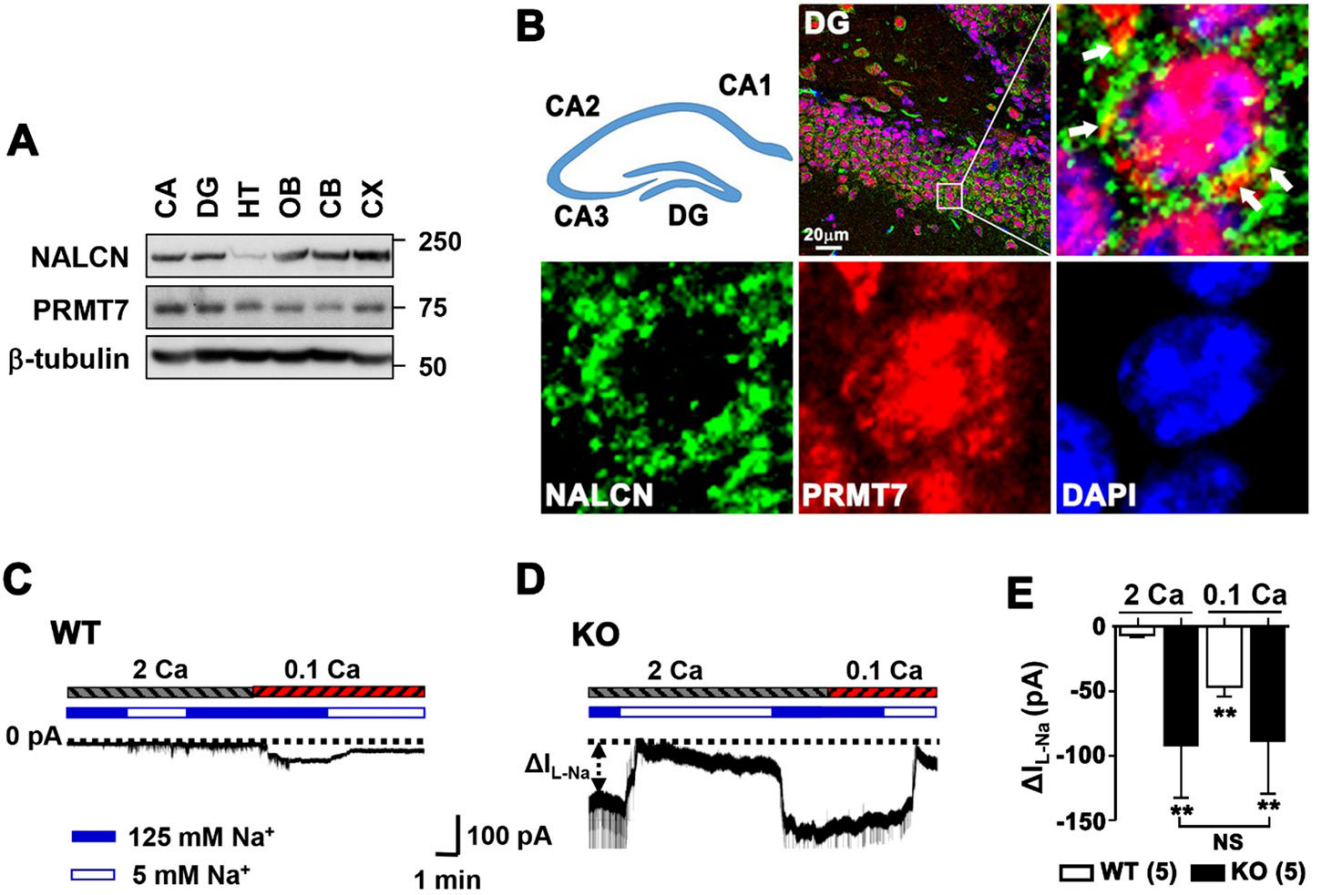
The sodium leak current I_L-Na_ is increased in KO granule cells. **a** Immunoblotting for NALCN and PRMT7 proteins in various brain regions, including CA and DG of the hippocampus. **b** Immunostaining of NALCN (green) and PRMT7 (red) in the DG region of a 1-month-old mouse brain. Scale bar = 20 µm. **c**, **d** Representative holding currents at -68 mV in dentate granule cells from WT (**c**) and KO mice (**d**). The dotted lines indicate the 0 current level. ΔI_L-Na_ (indicated by double-headed arrow) was calculated as the difference recorded between the 125 mM and 5 mM Na^+^-containing baths. ΔI_L-Na_ increased when [Ca^2+^]_e_ was switched from 2 mM (indicated by the hatched bar labeled 2 Ca) to 0.1 mM (0.1 Ca) in WT granule cells but not in KO granule cells. **e** Comparison of ΔI_L-Na_ between WT (*n* = 5) and KO granule cells (*n* = 5) at a range of [Ca^2+^]_e_, as indicated. **p < 0.01 compared to WT with 2 mM [Ca^2+^]_e_

In a search for the PRMT7 target, we examined SK channels, which are known to regulate the membrane excitability of granule cells^27^. The data obtained using apamin, a specific SK channel blocker, ruled out the contribution of these channels to the hyperexcitability of KO granule cells (SI Appendix, Fig. S3). We then tested the involvement of the Na^+^ leak channel, NALCN. The NALCN-mediated current is not blocked by TTX but can be reduced by either a nonspecific nonselective cation channel (NSCC) blocker Gd^3+^ or with the replacement of extracellular sodium with NMDG^18^, and it can be augmented by lowering extracellular Ca^2+^ levels^20^. We confirmed that NALCN protein is expressed in various brain areas, including the DG and CA of the hippocampus, similar to PRMT7 (Fig. 2a). The immunostaining of DG further showed that PRMT7 and NALCN are coexpressed and partially colocalized in granule cells (Fig. 2b, marked with white arrow). To assess the involvement of Na^+^ leak currents, currents were measured in the presence of TTX and Cs^+^ to block the contribution from Na_V_s and HCNs, respectively. We isolated the small Na^+^ leak current by measuring the difference (ΔI_L-Na_) between holding currents obtained in baths containing 125 mM and 5 mM Na^+^. This current increased from −5.6 ± 1.4 pA to −46.5 ± 7.0 pA (*p* < 0.01, *n* = 5) when [Ca^2+^]_e_ was lowered from 2 mM to 0.1 mM (Fig. 2c, e). Consistent with the currents generated by NALCN (I_NALCN_)^18^, the low [Ca^2+^]_e_-activated current was blocked by 10 μM Gd^3+^ (data not shown). Strikingly, KO granule cells exhibited large ΔI_L-Na_ (−92.2 ± 40.2 pA vs WT in control; −5.6 ± 1.4 pA; *p* < 0.01), which was not further increased when [Ca^2+^]_e_ was lowered to 0.1 mM (−88.7 ± 40.6 pA; *p* > 0.05, Fig. 2d, e). These data suggest that there is a small resting NALCN-mediated conductance in WT granule cells, and this conductance is dramatically enhanced in KO granule cells.

### Enhanced NALCN activity contributes to hyperexcitability in KO granule cells

**Fig. 3:**
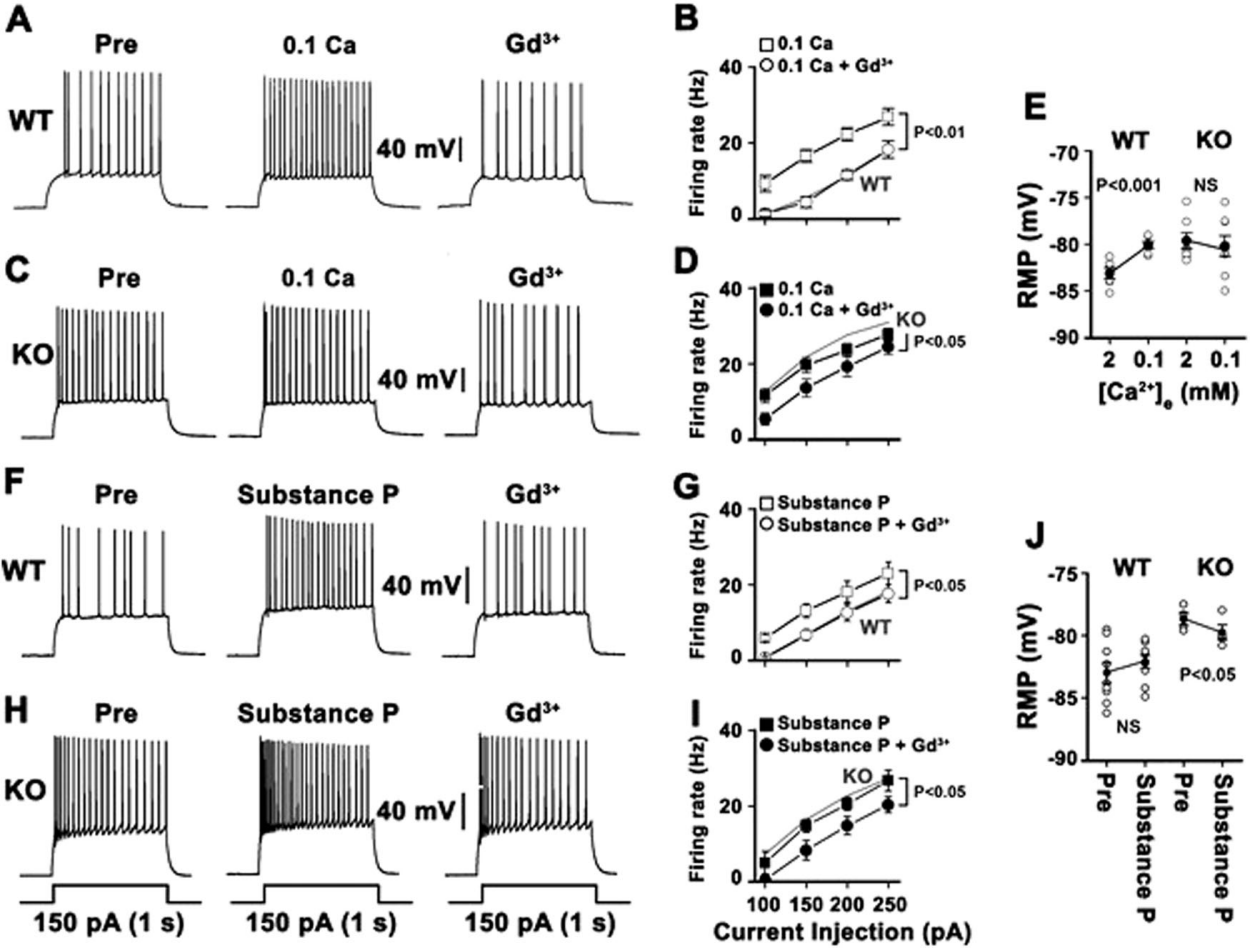
Enhanced NALCN activity contributes to hyperexcitability in KO granule cells. APs were evoked by applying 1-s depolarizing current pulses of different intensities (100–250 pA) to WT or KO granule cells. **a** and **c** illustrate representative traces in baths containing 2 mM (left traces) or 0.1 mM (middle traces) Ca^2+^ and the subsequent application of Gd^3+^. Summarized data compare the number of Aps before and after the application of Gd^3+^ in WT (**b**) and KO granule cells with 0.1 mM Ca^2+^ (**d**). The gray lines in B and D show the number of APs observed in WT and KO granule cells with 2 mM Ca^2+^, respectively. **e** The mean value of the resting membrane potential in baths containing 2 mM or 0.1 mM Ca^2+^. **f** and **h** illustrate representative traces before and after the application of substance P and the subsequent application of Gd^3+^. Summarized data compare the number of APs before and after the application of Gd^3+^ in WT (**g**) and KO granule cells with substance P(I). The gray lines in **g** and **i** show the number of APs observed in WT and KO granule cells, respectively, under control conditions. **j** The mean value of resting membrane potential before and after the application of substance P. Each dot represents an individual granule cell

Next, we assessed whether the altered NALCN currents contribute to neuronal hyperexcitability in KO granule cells (Fig. 3). Since specific NALCN inhibitors are not available, we initially used pharmacological tools that augment NALCN activities. NALCN is activated by a reduction in [Ca^2+^]_e_ or by treatment with substance P in hippocampal neurons^5,20^. Consistent with previous studies, in WT neurons, lowering [Ca^2+^]_e_ from 2 mM to 0.1 mM increased the firing frequencies elicited by depolarizing current injection (Fig. 3a, b) and induced a large depolarization of the RMP (3.0 ± 0.3 mV, *p* < 0.001, *n* = 6) (Fig. 3e). The low [Ca^2+^]_e_-induced excitatory effects were blocked by Gd^3+^ (Fig. 3a, b). In contrast, KO granule cells were not further excited by a drop in the [Ca^2+^]_e_ concentration to 0.1 mM (Fig. 3c, d). Interestingly, the application of Gd^3+^ recovered the firing frequency of KO granule cells to the level of WT cells. Given that NALCN is sensitive to inhibition by Gd^3+^, these data suggest that NALCN contributes to hyperexcitability in these neurons. Consistently, the depolarization observed in WT neurons by lowering [Ca^2+^]_e_ concentrations to 0.1 mM was largely absent in KO granule cells (*p* > 0.05, *n* = 7) (Fig. 3e). We also examined the effects of substance P on neuronal excitability in WT and KO granule cells. In WT granule cells that had small NALCN currents at basal conditions, firing frequency in response to a 150 pA depolarizing current was substantially increased from 6.9 ± 1.1 to 13.1 ± 1.8 Hz (*p* < 0.05, *n* = 9; Fig. 3f, g) by 1 μM substance P. This response in WT granule cells was blocked by Gd^3+^. If NALCN is already active in KO granule cells, substance P treatment might exert a small effect. Consistent with this prediction, in KO granule cells, substance P did not further increase the firing frequency, and the subsequent application of Gd^3+^ reduced the firing frequency to the level of WT granule cells (Fig. 3h). Furthermore, in KO granule cells, substance P application induced membrane hyperpolarization rather than depolarization (*n* = 4, *p* < 0.05; Fig. 3j). These data suggest that the NALCN-mediated current is increased in KO granule cells, contributing to neuronal hyperexcitability.

### PRMT7 depletion enhances NALCN activity by increasing its sensitivity to [Ca^2+^]_e_

**Fig. 4:**
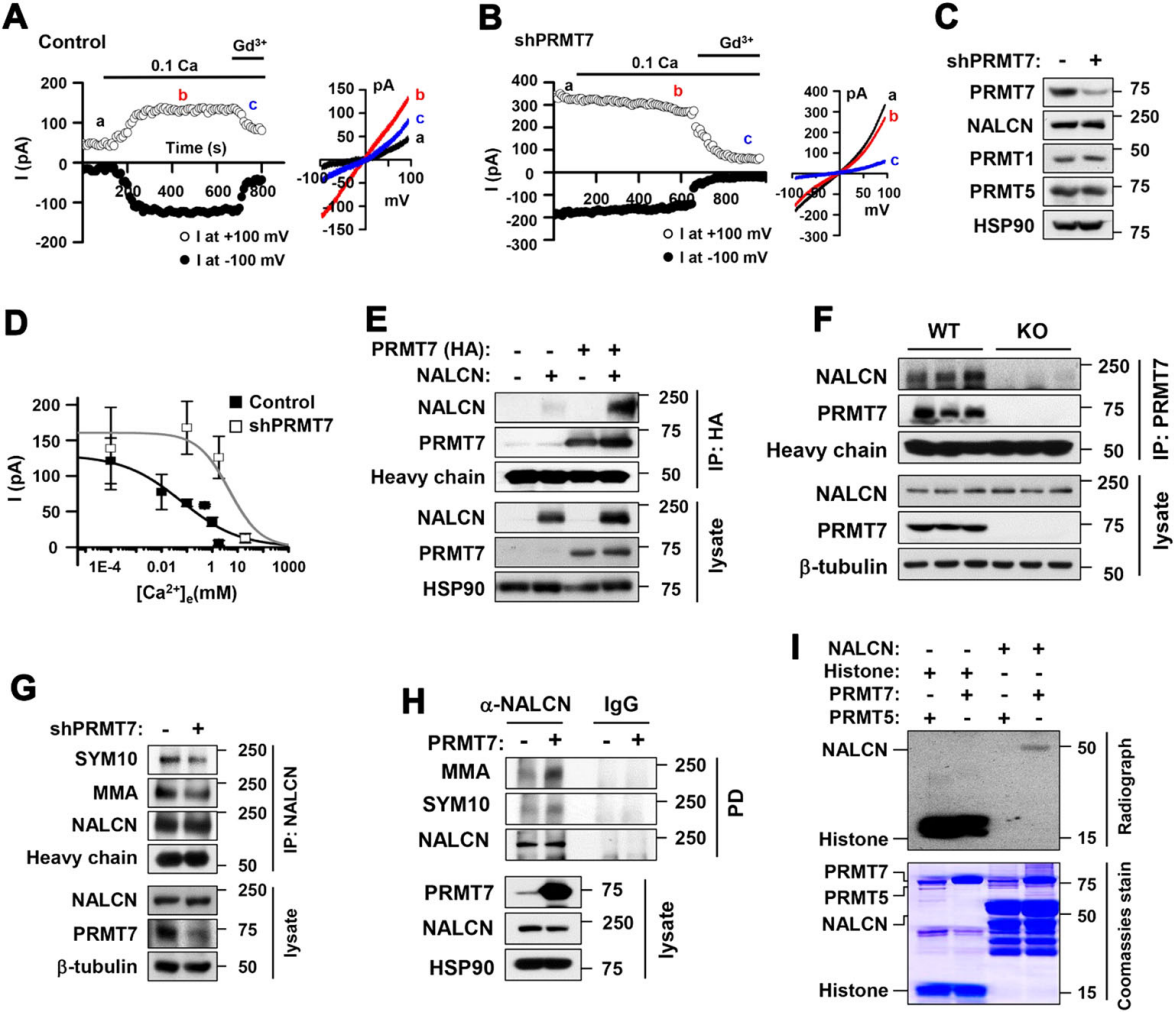
PRMT7 regulates NALCN activity through arginine methylation. Recordings from HEK293T cells expressing NALCN with the control vector (**a**) or a PRMT7 shRNA (**b**) in response to 0.1 mM Ca^2+^ and the subsequent application of Gd^3+^. Inset, I-V relationships were obtained from the data to the left. **c** Control immunoblotting for PRMT7 expression. **d** Dose-response relationship of NALCN currents in response to changes in [Ca^2+^]_e_ in WT and PRMT7 knockdown cells. **e** Immunoprecipitation analysis of HEK293T cells transfected with HA-tagged PRMT7 (HA-PRMT7) and rat NALCN expression vector. Cell lysates were immunoprecipitated with anti-HA antibodies and immunoblotted with anti-NALCN or anti-HA antibodies. **f** Immunoprecipitation analysis of whole mouse brain lysates from 2-month-old mice with anti-PMRT7 antibodies, followed by immunoblotting with the indicated antibodies. **g** Immunoprecipitation analysis of HEK293T cells transfected with shPRMT7 or control expression vectors. Cell lysates were immunoprecipitated with anti-NALCN antibodies followed by immunoblotting with anti-dimethyl-arginine, symmetric (SYM10), anti-mono-methyl-arginine (MMA) or NALCN antibodies. **h** Immunoprecipitation analysis of HEK293T cells transfected with HA-PRMT7 or control expression vectors. Cell lysates were immunoprecipitated with anti-NALCN antibodies or mouse IgG as a control, followed by immunoblotting with the indicated antibodies. **i** Autoradiography for in vitro methylation assay using immunoprecipitated HA-PRMT5, HA-PRMT7 and bacterially purified GST-NALCN (1588-1713). Purified total histone was used as a positive control

To investigate the detailed mechanism, the effect of PRMT7 knockdown on NALCN current was examined. We reconstituted NALCN currents in HEK293T cells, which do not have significant endogenous leak currents, and measured the channel function by applying a ramping protocol from −100 mV to +100 mV in 1-s at a holding potential of 0 mV. Based on previous reports, NALCN activity is regulated by the Src family of tyrosine kinases (SFKs) in neurons^28^. To reconstitute the NALCN current in a heterologous expression system such as HEK293 cells, the coexpression of a constitutively active Src (Src529) with other obligatory NALCN and accessory proteins is required to increase the percentage of cells expressing detectable NALCN current^18,28^. Thus, we cotransfected NALCN with Src529 and with two other accessory proteins, mUNC-80 and CaSR, in HEK293 cells to reconstitute NALCN currents. Consistent with previous studies^20^, control HEK293T cells expressing NALCN, mUNC-80, active Src, and CaSR activated a current (12.0 ± 2.0 pA/pF at +100 mV; Fig. 4a) with a linear I–V relationship in the condition lowering [Ca^2+^]_e_ from 2 mM to 0.1 mM (Fig. 4a, inset). Like NALCN currents in neurons, the current was blocked by Gd^3+^. Compared to control cells, PRMT7-knockdown cells exhibited remarkably increased basal currents. The current density at +100 mV at 2 mM of [Ca^2+^]_e_ was 43.3 ± 3.3 pA (*n* = 17) and 179.3 ± 33.4 pA (*n* = 9, *p* < 0.01) for control and PRMT7 knockdown cells, respectively. These currents were not further activated by a reduction in [Ca^2+^]_e_ and were blocked by Gd^3+^, suggesting that these increased leak currents are NALCN-dependent. The potentiation of currents by low [Ca^2+^]_e_ was 120.4 ± 28.5% (*n* = 6; Fig. 4a) and −2.7 ± 5.6% (*n* = 6, *p* < 0.01 vs control; Fig. 4b) for control and PRMT7*-*depleted cells, respectively. Importantly, NALCN protein levels were unchanged by PRMT7 depletion, suggesting that increased NALCN activities related to PRMT7 depletion might be due to alterations in the functional properties of NALCN (Fig. 4c). Thus, we examined whether PRMT7 shifts the NALCN currents’ sensitivity to [Ca^2+^]_e_. In control-transfected cells, the Na^+^ leak current faithfully reflected [Ca^2+^]_e_ changes in a wide range of concentrations between 0.0001 and 20 mM (Fig. 4d, closed square); lowering [Ca^2+^]_e_ increases NALCN currents. PRMT7 depletion reduced the [Ca^2+^]_e_ sensitivity of NALCN, shifting the dose-response curve to the right (Fig. 4d, open square; IC50 = 0.06 ± 0.12 mM (*n* = 17) for control and 4.55 ± 5.1 mM (*n* = 9) for PRMT7-knockdown cells). Taken together, these data suggest that PRMT7 regulates NALCN channel activity via its [Ca^2+^]_e_ sensitivity.

### PRMT7 interacts with and methylates NALCN

To examine the molecular mechanism by which PRMT7 regulates NALCN activity, we examined a potential interaction between PRMT7 and NALCN. HEK293T cells transfected with PRMT7 and/or NALCN were subjected to coimmunoprecipitation (Fig. 4e). NALCN and PRMT7 proteins were coprecipitated when coexpressed, and the endogenous interaction was further confirmed in mouse hippocampal lysates (Fig. 4f). Consistent with the result from PRMT7-knockdown cells, NALCN levels in KO brains did not differ from those in WT brains. Thus, we assessed whether PRMT7 methylates NALCN to modulate its activity. PRMT7 catalyzes the mono- and symmetric di-methylation of arginine residues in substrates^29,30^. HEK293T cells expressing NALCN were transfected with control or PRMT7 shRNA, followed by NALCN immunoprecipitation and immunoblotting with antibodies recognizing mono-methylated (MMA) or symmetrically di-methylated arginine (SYM10). In control cells, the MMA- and SYM10-specific NALCN proteins were readily detected, while PRMT7 knockdown substantially decreased both types of methylated NALCN levels (Fig. 4g). Conversely, PRMT7 overexpression enhanced the level of mono- and symmetric di-methylated NALCN (Fig. 4h). These data suggest that PRMT7 can methylate NALCN protein. It has been reported that the mechanism by which [Ca^2+^]_e_ regulates NALCN is CaSR-dependent and involves molecular determinants in the carboxy (C)-terminal domain of NALCN^2,20^. Since it appears that NALCN hyperactivity in PRMT7-deficient granule cells is associated with defects in the calcium sensing function of NALCN, we examined whether the C-terminal domain of NALCN is the target of PRMT7. To do this, we generated a bacterial expression vector containing the GST-tagged C-terminal region of NALCN from 1588 to 1713, and purified proteins were used for in vitro methylation assays with purified HA-tagged PRMT7 and PRMT5 as controls (Fig. 4i). Both PRMTs methylated control histone substrates, while GST-NALCN was methylated by PRMT7 but not by PRMT5. Taken together, these data suggest that PRMT7 interacts with and methylates NALCN in its C-terminal domain.

### The substitution of arginine 1653 to lysine led to enhanced NALCN activity

**Fig. 5:**
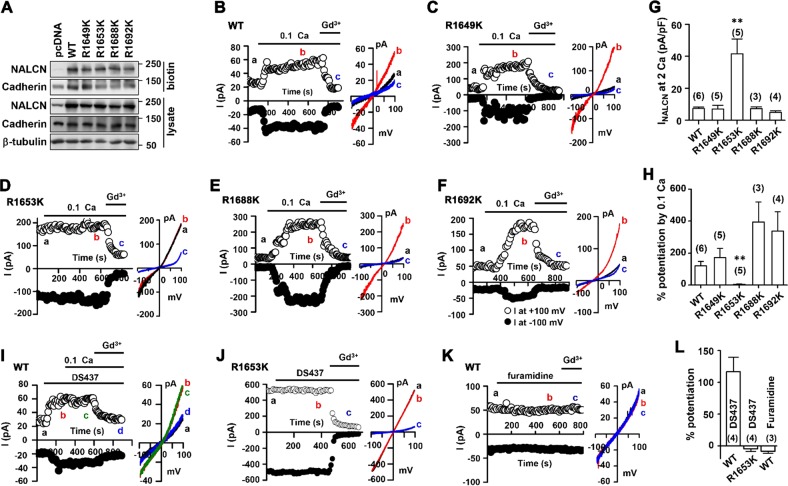
The substitution of arginine 1653 with lysine led to enhanced NALCN activity. **a** Surface biotinylation/pull down assay from HEK293T cells transfected with control, NALCN WT or point mutation protein. The arginine residue at 1649, 1653, 1688 or 1692 in rat NALCN was changed to lysine (R1649K, R1653K, R1688K, and R1692K). **b**–**f** Time course for the effect of 0.1 mM [Ca^2+^]_e_ on NALCN current in HEK293T cells expressing WT NALCN (**b**), R1649K (**c**), R1653K (**d**), R1688K (**e**) or R1692K (**f**) mutants. NALCN currents were obtained with a voltage-ramp protocol (−100 mV to +100 mM in 1 s). Inset, I-V relationships were obtained from the data to the left. Panels G and H illustrate the current density of NALCN at +100 mV for WT and each mutant (**g**) and the percentage of the potentiation of NALCN current by 0.1 mM [Ca^2+^]_e_ (**h**). A PRMT7 inhibitor, DS437 (0.1 mM), was applied during the periods indicated by bars in WT (**i**) and R1653K mutant (**j**). **k** The inhibition of PRMT1 with a specific inhibitor, furamidine had no effect on WT NALCN currents. **l** Summarized data for NALCN activities with DS437 and furamidine

The sequence analysis of the C-terminal domain of NALCN revealed six arginines as potential targets for PRMT7. Thus, we generated expression vectors for NALCN with a substitution of arginine to lysine (referred to as RK mutants) at amino acid positions of 1649, 1653, 1664, 1670, 1688, or 1692, which have not been characterized by other studies. Although R1649K, R1653K, R1688K, or R1692K mutants were well expressed in HEK293T cells, similar to WT NALCN (Fig. 5a), we were unable to express R1664K and R1670K proteins, and the underlying reason for this inability is currently unclear. Next, the activity of WT cells and R1649K, R1653K, R1688K, and R1692K mutants was assessed using the conventional whole-cell patch clamp technique. The currents of RK mutants are shown in Fig. 5b–f. Compared to WT, the R1653K mutant exhibited remarkably increased currents that were not further activated by a reduction in [Ca^2+^]_e_ but were blocked by Gd^3+^. The current density at +100 mV was 7.5 ± 0.8 pA/pF (*n* = 6) and 41.6 ± 9.2 pA/pF (*n* = 5, *p* < 0.01 vs WT) for WT and R1653K, respectively (Fig. 5g). The potentiation of currents by low [Ca^2+^]_e_ was 120.6 ± 28.6% (*n* = 6) and 5.8 ± 1.8% (*n* = 5, *p* < 0.01 vs WT) for WT and R1653K, respectively (Fig. 5h). Other RK mutant-expressing cells (R1649, R1688, and R1692) showed leak currents undistinguishable from those of WT cells with a current density (pA/pF) at +100 mV of 7.2 ± 2.2 pA/pF (*n* = 5, *p* > 0.05), 7.3 ± 1.2 pA/pF (*n* = 3, *p* > 0.05), and 5.1 ± 1.0 pA/pF (*n* = 4, *p* > 0.05), respectively (Fig. 5g). In addition, mutant-expressing cells had similar [Ca^2+^]_e_-induced potentiation to that of WT (Fig. 5h). In summary, these data suggest that the arginine residue at 1653 of NALCN is important for the control of NALCN activities.

To further confirm this result, we examined the effect of PRMT7 inhibition by DS437 on NALCN currents. As shown in Fig. 5i, DS437 dramatically increased WT NALCN currents, which was not further increased by lowering [Ca^2+^]_e_. The NALCN currents were confirmed by Gd^3+^ treatment. However, DS437 did not augment the activities of R1653K (Fig. 5j), indicating R1653 as the target site for PRMT7. In contrast, PRMT1 inhibition with furamidine did not affect NALCN currents (Fig. 5k), suggesting the specificity of PRMT7 in NALCN regulation. DS437 or furamidine increased WT NALCN activities by 116.5 23.2% (*n* = 4) and −8.7 ± 3.1% (*n* = 3), respectively (Fig. 5l). Taken together, our data suggest that the PRMT7-mediated methylation of NALCN at R1653 suppresses its channel activity.

### Arginine methylation of NALCN at R1653 facilitates its phosphorylation at S1652, contributing to NALCN channel inhibition

**Fig. 6:**
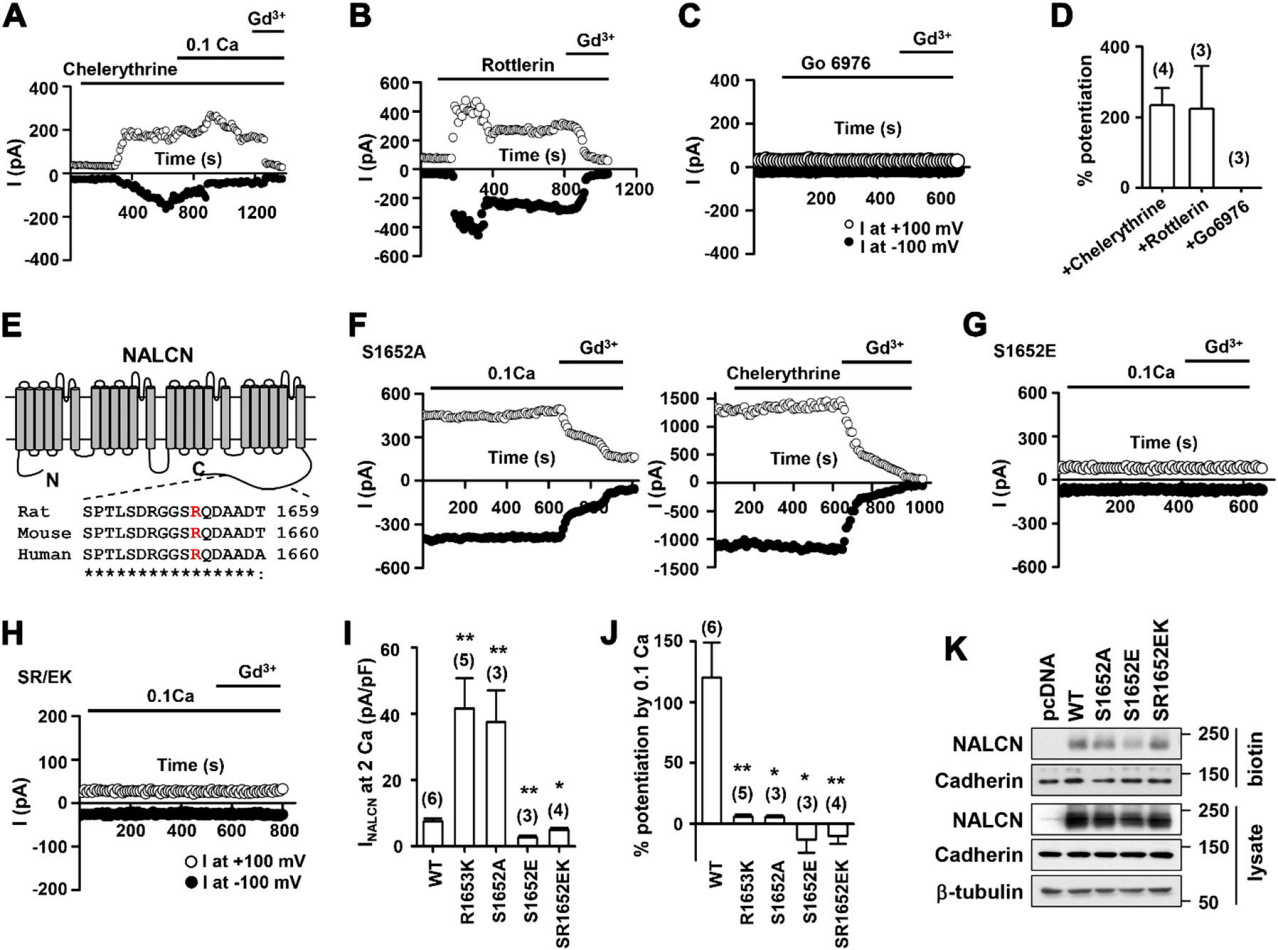
Arginine methylation of NALCN is required for CaSR-mediated NALCN control. **a**–**c** Time course for the effect of chelerythrine (50 μM, **a**), rottlerin (1 μM, **b**) and Go 6976 (10 μM, **c**) on NALCN activities in HEK293T cells. **d** Summary of NALCN potentiation by each PKC inhibitor. **e** Schematic illustration of the conserved amino acid of the NALCN C-terminal (from the rat, mouse, human isoform) is shown. **f**–**h** Time course for the effect of 0.1 mM [Ca^2+^]_e_ and chelerythrine on NALCN currents in HEK293T cells expressing S1652A (**f**), S1652E (**g**) and SR/EK (**h**). **i**, **j** The current density of NALCN at +100 mV for WT and each mutant is summarized in (**i**), and the percentage of potentiation of the NALCN current by 0.1 mM [Ca^2+^]_e_ is shown in (**j**). **k** Surface biotinylation pull down assay from HEK293T cells transfected with control, NALCN WT or mutants. The serine residue at 1652 in rat NALCN was changed to alanine or glutamic acid (S1652A, S1652E), while the double point mutation at residues serine 1652 and arginine 1653 were switched to glutamic acid or lysine (SR1652EK), respectively

Given that PRMT7 alters NALCN’s sensitivity to [Ca^2+^]_e_, it is plausible that PRMT7 regulates NALCN channel activity through CaSR. CaSR suppresses NALCN activity in a G-protein-dependent manner^20^. Furthermore, the CaSR-dependent regulation of basolateral K^+^ channels is mediated via PLC–PKC pathways in kidney cells^31^. Thus, we tested the relationship between PRMT7 and the CaSR/PKC pathway in the control of NALCN activity. To do this, the effect of chelerythrine, a general inhibitor of classical and novel PKC, on NALCN current was examined. Chelerythrine activated NALCN currents in baths containing 2 mM Ca^2+^ (18.9 ± 3.2 pA/pF at +100 mV, *n* = 4; Fig. 6a, d), indicating that it abrogated the CaSR-mediated inhibition of NALCN. Similarly, rottlerin, a selective inhibitor of the novel PKC-delta isoforms, activated NALCN currents (20.1 ± 8.7 pA/pF at +100 mV, *n* = 3; Fig. 6b, d). In contrast, Go 6976, which selectively inhibits classic PKCα and PKCβ1-isoforms, had little effect on NALCN currents (Fig. 6c, d). Furthermore, a PKC activator, PMA, suppressed low [Ca^2+^]_e_-induced NALCN currents (SI Appendix, Fig. S4). Collectively, these data suggest that novel PKC isoforms, likely PKC delta, are part of downstream signaling in CaSR-mediated NALCN inhibition.

The arginine residue at 1653 of rat NALCN is highly conserved across species, including mice and humans (Fig. 6e). Interestingly, we noticed that the serine residue at 1652 adjacent to R1653 in NALCN proteins is predicted as a potential target of PKC by the kinase-specific phosphorylation site prediction program (http://www.cbs.dtu.dk/services/NetPhos/). Thus, we hypothesized that this serine residue is a potential target of the CaSR/PKC pathway. To test this possibility, we generated NALCN mutants harboring a substitution of serine 1652 to alanine (a phosphorylation-deficient SA mutant, S1652A) or a substitution of serine to glutamate (a phsophomimetic SE mutant, S1652E). Similar to R1653K, the channel activities of S1652A were significantly increased compared to those of the WT channel (37.5 ± 9.6 (*n* = 3) pA/pF, *p* < 0.01 vs WT) and were insensitive to lowering [Ca^2+^]_e_ (5.6 ± 1.0% (*n* = 3), *p* < 0.05 vs WT) (Fig. 6f). Furthermore, S1652A did not exhibit increasing effects in response to chelerythrine (Fig. 6f). In contrast, the expression of the phosphomimetic mutant S1652E did not augment channel activity when the [Ca^2+^]_e_ was lowered to 0.1 mM (Fig. 6g, i, j), suggesting that the dephosphorylation of serine 1652 is required for the release of NALCN from CaSR-mediated inhibition. To gain further insights, we generated the double mutant, S1652E/R1653K. S1652E/R1653K exhibited basal currents and [Ca^2+^]_e_ sensitivities similar to those in the S1652E single mutant, suggesting that the arginine methylation of R1653 facilitates the phosphorylation of S1652 leading to channel inhibition (Fig. 6h). The data are summarized in Fig. 6i, j.

To examine whether the phosphorylation of NALCN modulates its surface localization, HEK293T cells expressing control, WT, S1652A, S1652E or S1652E/R1653K proteins were subjected to surface biotinylation experiments. Interestingly, there was no relevant difference in the surface localization of NALCN mutants compared to that of WT protein (Fig. 6k). The expression of S1652A and S1652E/R1653K mutants was similar to that of WT proteins, and the S1652E mutant was slightly lower overall, as well as having lower surface levels. Considering that S1652E and S1652E/R1653K have similar effects on channel function, however, the slight reduction in the expression level of S1652E might be irrelevant to the alteration of functional properties in this mutant. These data suggest that the phosphorylation of NALCN regulates channel activity by mechanisms other than the modulation of membrane targeting of channel proteins. Taken together, these data demonstrate a novel regulatory mechanism of NALCN channel activity by PRMT7. PRMT7 interacts with and methylates NALCN at arginine residue 1653, thereby negatively regulating its activity by promoting the phosphorylation of serine residue 1652. Our working hypothesis proposes that when PRMT7 is depleted or its activity is inhibited, demethylated NALCN is relieved from CaSR-mediated inhibition, contributing to neuronal hyperexitability (SI Appendix, Fig. S5).

## Discussion

In this study, we describe a novel regulatory mechanism whereby PRMT7 regulates NALCN activities and the overall excitability of hippocampal DG granule cells. Using a combination of genetic, biochemical, and electrophysiological approaches, our work reveals a molecular pathway that links PRMT7 to NALCN regulation. PRMT7 knockout granule cells exhibited hyperexcitability due to the perturbation of NALCN activity. PRMT7 knockdown by shRNA in HEK293T cells confirmed the in vivo results and showed that PRMT7 depletion increased NALCN activity by shifting the dose-response curve of NALCN inhibition by [Ca^2+^]_e_ without affecting NALCN protein levels. Our molecular analysis demonstrated that PRMT7 binds to and methylates arginine residue 1653 in the C-terminal region of NALCN. Interestingly, the C-terminal region of NALCN has been shown to play a critical role in the sensitivity of NALCN to [Ca^2+^]_e_^20^. Consistent with this result, the experiment using the methylation-deficient mutant NALCN R1653K further supports that this highly conserved arginine is indeed required for the sensitivity of NALCN to [Ca^2+^]_e_.

Although the detailed molecular regulatory mechanism of NALCN activity is still largely uncharacterized, CaSR is known to be an important regulator of NALCN activity. CaSR is widely expressed in the brain and has emerged as an important signaling pathway in neurological diseases such as Alzheimer’s disease^32^. In the brain, extracellular Ca^2+^ levels can fall sharply during neuronal activity^33,34^. CaSR transduces changes in [Ca^2+^]_e_ into increased neuronal excitability, modulating some neuronal channels, including NALCN and Ca^2+^-activated K^+^ channels^20,34^. Based on the result showing that the activity of SK channels, a major Ca^2+^-activated K^+^ channel, is unaltered in KO granule cells (SI Appendix, Fig. S2), we can rule out the contribution of the SK channel to this phenotype. CaSR-mediated NALCN modulation remains incompletely understood except for the fact that it involves G protein^30^. However, CaSR’s effects on other biological processes can be mediated via classic or novel PKC pathways^35,36^. Thus, we tested the involvement of PKC in NALCN activity control in relation to PRMT7-mediated arginine methylation. Among the tested PKC inhibitors on NALCN activity, rottlerin, an inhibitor of PKC delta but not classic PKC, increased NALCN activity in a bath containing 2 mM Ca^2+^. Although the IC50 for chelerythrine and Go6976 are 0.66 μM^37^ and 20 nM^38^, respectively, we used higher concentrations of chelerythrine and Go6976 than the IC50 (50 μM for chelerythrine and 10 μM for Go6976) in the current study. However, likely due to the short period of treatment via fast superfusion, patch clamp studies such as our current study frequently use inhibitors with concentrations more than 10 times higher than the IC50^39–41^. We found that chelerythrine, a general inhibitor of classical PKC and novel PKC, activated NALCN currents, while Go6976, a selective inhibitor of PKC alpha and PKC beta1, had little effect even at this high concentration. These data suggest that novel PKC isoforms might regulate NALCN activities. Since PKC isoenzymes are differentially inhibited depending on the concentration of rottlerin (IC50 values: 3–6 μM for PKC delta, 30–42 μM for PKC alpha, beta, gamma, 80–100 μM for PKC epsilon, eta, zeta)^42^, we tested the variable concentrations of rottlerin. Rottlerin at 1 μM affected NALCN currents, which is close to IC50 for PKC delta; thus, we concluded that PKC delta might be important for NALCN regulation.

The involvement of PKC delta in CaSR-mediated NALCN has not been reported until now. However, it has been previously shown that CaSR mediates cardiac apoptosis via the activation of PKC delta^36^. Using the sequence prediction program, we identified S1652 in NALCN, adjacent to the R1653 methylation site, as a potential PKC phosphorylation site. The analysis with the phosphorylation- and/or methylation-defective NALCN mutants demonstrated that the phosphorylation of S1652 is critical for the suppression of channel activity and that the methylation of NALCN at R1653 facilitates this phosphorylation. Taken together, we propose that NALCN phosphorylation is an on/off switch for the activity of NALCN and that this phosphorylation is delicately controlled by the cooperation of PRMT7 and CaSR (SI Appendix, Fig. S5). PRMT7 ablation from dentate granule cells decreases NALCN methylation at R1653, which, in turn, decreases CaSR/PKC-mediated NALCN phosphorylation at S1652, lifting NALCN inhibition, thereby enhancing neuronal excitability. Given that the treatment of WT hippocampal slices with the PRMT7 inhibitor DS437 for 10 min can induce neuronal hyperexcitability similar to that induced by PRMT7 deficiency, methylation at R1653 by PRMT7 might be a dynamic process modulating channel activity. Further work is required to determine whether the control of NALCN by PRMT7 is broadly applied in NALCN-expressing cells such as suprachiasmatic nucleus (SCN) pacemaker neurons^43^ or substantia nigra pars reticulata (SNr) neurons^44^.

The study of the regulatory mechanisms of intrinsic neuronal excitability has largely focused on the modulation of resting potassium conductance^14,27,45^. Surprisingly, we observed an increase in sodium leak conductance in KO granule cells that is mediated by NALCN channels. This sodium leak exhibits the pharmacological sensitivity previously defined for the NALCN current^18^, most notably to NMDG and Gd^3+^ block. In addition, no further increase in excitability was observed in KO granule cells with the reduction in [Ca^2+^]_e_ levels or with the application of substance P, both of which elicit excitatory effects via NALCN, while Gd^3+^ recovered excitability to control levels, indicating that increased NALCN currents contribute to hyperexcitability in KO granule cells. A recent study employing the conditional knockout of NALCN in forebrain excitatory neurons demonstrated that NALCN is necessary for the appropriate excitability of SCN neurons and underlies circadian rhythms in flies and rodents^43^. In addition, NALCN is reported to play a key role in spontaneously firing GABAergic SNr neurons^44^. Interestingly, we observed little NALCN-induced leak activity in WT granule cells or in HEK293T cells. Thus, it is plausible that different types of neurons display distinct basal NALCN activities depending on the activity strength of GPCRs or PRMT7, which, in turn, determines the inherent activity level of neurons as their intrinsic excitability. It is known that the basal activity of GPCRs can be modulated by factors such as their binding partners^46,47^. Consistent with this result, Swayne et al. reported that there is little NALCN activity at the basal level, which can be activated by M3 muscarinic receptors (M3R) in a pancreatic β-cell line^48^.

Together with a previous study, our data imply that PRMT1 and PRMT7 target distinct classes of ion channels to control neuronal function. PRMT1 controls membrane excitability via the KCNQ K^+^ channel^14^ and does not regulate NALCN activity. In contrast, PRMT7 regulates NALCN activity without affecting KCNQ K^+^ channel activity. Both NALCN and KCNQ channels contribute to the RMP of neurons and control neuronal function, but in opposite directions; NALCN depolarizes neurons and increases intrinsic excitability, while KCNQ channels oppose sustained membrane depolarization and repetitive AP firing^14,49^. Thus, PRMT1 and PRMT7 collaborate to control intrinsic excitability in hippocampal neurons by increasing the repolarizing drive and suppressing the opposite depolarizing drive, respectively. In summary, we have uncovered a new signaling link between PRMT7 and NALCN in the control of neuronal excitability, thus providing a mechanism for the prevention of neuronal hyperexcitability via sodium conductance.

## Supplementary information

Supplementary information
