# Supplementary material for: Methylation determines the extracellular calcium sensitivity of the leak channel NALCN in hippocampal dentate granule cells
Source: Exp Mol Med. 2019 Oct 10;51(10):119. doi: 10.1038/s12276-019-0325-0 (PMC6802672; doi:10.1038/s12276-019-0325-0)
Supplement: Supplementary file 1 — Supplementary information [file 12276_2019_325_MOESM1_ESM.docx]

| **Supplementary Table1. List of antibodies used in this study** | | |  |
| --- | --- | --- | --- |
| Antibodies | Species | Company | Rate |
| β-tubulin | Mouse | Sigma (T5293) | 1:2000 (WB) |
| HSP90 | Rabbit | Santa Cruz (H-114, SC-7947) | 1:1000 (WB) |
| NALCN | Mouse | Sigma (SAB 5200672) | 1:200(IF), 1:1000(WB) |
|  | Rabbit | Gentex (GTX 54808) | 1:200(IF), 1:1000(WB) |
| PRMT7 | Goat | Santa Cruz (N-17, sc-48660) | 1:200(IF), 1:1000(WB) |
|  | Rabbit | GeneTex (GTX116570) | 1:400(IF), 1:1000(WB) |
| PRMT1 | Rabbit | Millipore (07-404) | 1:1000 (WB) |
| PRMT5 | Rabbit | Cell signaling (2252S) | 1:1000 (WB) |
| pan-Cadherin | Mouse | abCam (ab6528) | 1:1000 (WB) |
| SYM10 | Rabbit | Millipore (07-412) | 1:500 (WB) |
| MMA | Rabbit | Cell signaling (8015) | 1:500 (WB) |

| **Supplementary Table2. List of primer used to generate expression vectors in this study** | | |
| --- | --- | --- |
| Vector | Primer | Sequence |
| pCMV2-rNALCN R1625K | Forward | GCCCTACTCTGTCAGACA**A**AGGAGGAAGTCGACAGG |
|  | Reverse | CCTGTCGACTTCCTCCT**T**TGTCTGACAGAGTAGGGC |
| pCMV2-rNALCN R1629K | Forward | GTCAGACAGAGGAGGAAGT**AA**ACAGGATGCAGCAGATACTG |
|  | Reverse | CAGTATCTGCTGCATCCTGT**TT**ACTTCCTCCTCTGTCTGAC |
| pCMV2-rNALCN R1664K | Forward | GTGTCTTCGGTTAACCTA**AA**GTTTGGAGGAAGGACAAC |
|  | Reverse | GTTGTCCTTCCTCCAAAC**TT**TAGGTTAACCGAAGACAC |
| pCMV2-rNALCN R1668K | Forward | CCTACGGTTTGGAGGAA**A**GACAACAATGAAGTCTG |
|  | Reverse | CAGACTTCATTGTTGTC**T**TTCCTCCAAACCGTAGG |
| pCMV2-rNALCN S1652A | Forward | CTGTCAGACAGAGGAGGA**GC**TCGACAGGATGCAGCAG |
|  | Reverse | CTGCTGCATCCTGTCGA**GC**TCCTCCTCTGTCTGACAG |
| pCMV2-rNALCN S1652E | Forward | CTCTGTCAGACAGAGGAGGA**GAA**CGACAGGATGCAGCAGATAC |
|  | Reverse | GTATCTGCTGCATCCTGTCG**TTC**TCCTCCTCTGTCTGACAGAG |
| pCMV2-rNALCN SR1652EK | Forward | CTCTGTCAGACAGAGGAGGA**GAAAA**ACAGGATGCAGCAGATACTG |
|  | Reverse | CAGTATCTGCTGCATCCTGT**TTTTC**TCCTCCTCTGTCTGACAGAG |
| pVFT2S-rNALCN 1588-1713 | F-EcoRI | TAGC GAATTC AGC ATT GAG ACC ACC CAA CC |
|  | R-XhoI | GCTA CTCGAG CTAGATATCTAGGAGGTCATC |

**Supplementary Figure legends**

**Fig. S1. Pharmacological inhibition of PRMT7 with DS437 for 10 min, increases intrinsic excitability in WT hippocampal granule cells.**

(**A**) Representative trace in the whole-cell current-clamp recording from WT granule cells before (left) and after 100 μM DS437 (middle) in response to 1-s depolarizing current injection (150 pA). (Right) the mean number of action potentials (AP No.) plotted against the eliciting currents (from 100 pA to 250 pA, + 50 pA increment, during 1-s). At all amplitudes, the mean ± S.E.M. Firing frequency in DS437-treated WT granule cells (■; n = 4) increased to the level of KO granule cells (gray line). (**B-G**) the mean value of resting membrane potential (**B**), threshold current for AP generation (100 ms duration; **C**), input resistance (**D**), threshold potential (**E**), AP height (**F**), and AP half-width (**G**) before and after treatment with 100 μM DS437 in WT granule cells.

**Fig. S2. PRMT7 is not involved in regulation of KCNQ2/3 currents.** (**A**) Representative traces were elicited by voltage steps from -60 mV to +30 mV (in 10 mV increments) with a subsequent step to -60 mV before and after treatment with DS437 (100 μM) and KCNQ channel blocker XE991 (50 μM) in HEK293T cells expressing KCNQ2/3 channels. (**B**) Percentage of inhibitions of KCNQ2/3 currents by furamidine or DS437. (**C, E**) Representative traces of whole-cell current-clamp recording in response to 1-s depolarizing current injection (150 pA) before and after 50 μM XE991 in dentate granule cells of WT (**C**) and KO granule cells (**E**). (**D, F**) The mean number of AP No. plotted against the eliciting currents (from 100 pA to 200 pA, + 50 pA increment, during 1-s) from WT (**D**) and KO granule cells (**F**). At all amplitudes, the mean ± S.E.M.

**Fig. S3. SK channel activity was not changed in KO granule cells, compared with WT granule cells.**

(**A-B**) Representative traces of whole-cell current clamp recordings from WT (**A**) and KO GC (**B**) showing repetitive spiking in response to a depolarizing current pulse (100-ms long) before and after bath application of 100 nM apamin. The background membrane potential prior to stimulation was kept at -62 mV (dashed line) by depolarizing holding current injection, and the current pulse amplitude was adjusted to produce a train of eight APs. The insets show expanded traces. (**C**) The summary graph shows the effect of apamin on the medium afterhyperpolarization (mAHP) in all cells tested (○) and the mean value (●) in WT and KO granule cells. The mAHP was not significantly different between WT (n=4) and KO granule cells (n=4). (NS, p>0.05).

**Fig. S4. A PKC activator, PMA suppressed the NALCN activities.** (**A**) Inhibition of the low [Ca^2+^]_e_-activated NALCN currents by 1 μM PMA in HEK293T cells. (**B**) Summarized data for the inhibition of NALCN currents by 1 μM PMA (n=3).

**Fig. S5. A working model.**

Based on our data, we propose a working hypothesis that PRMT7 methylates NALCN at arginine 1653 which is required for NALCN phosphorylation at S1652 by Ca^2+^-sensing receptor (CaSR). This regulatory mechanism might contribute to the prevention of neuronal hyperexcitability.

**
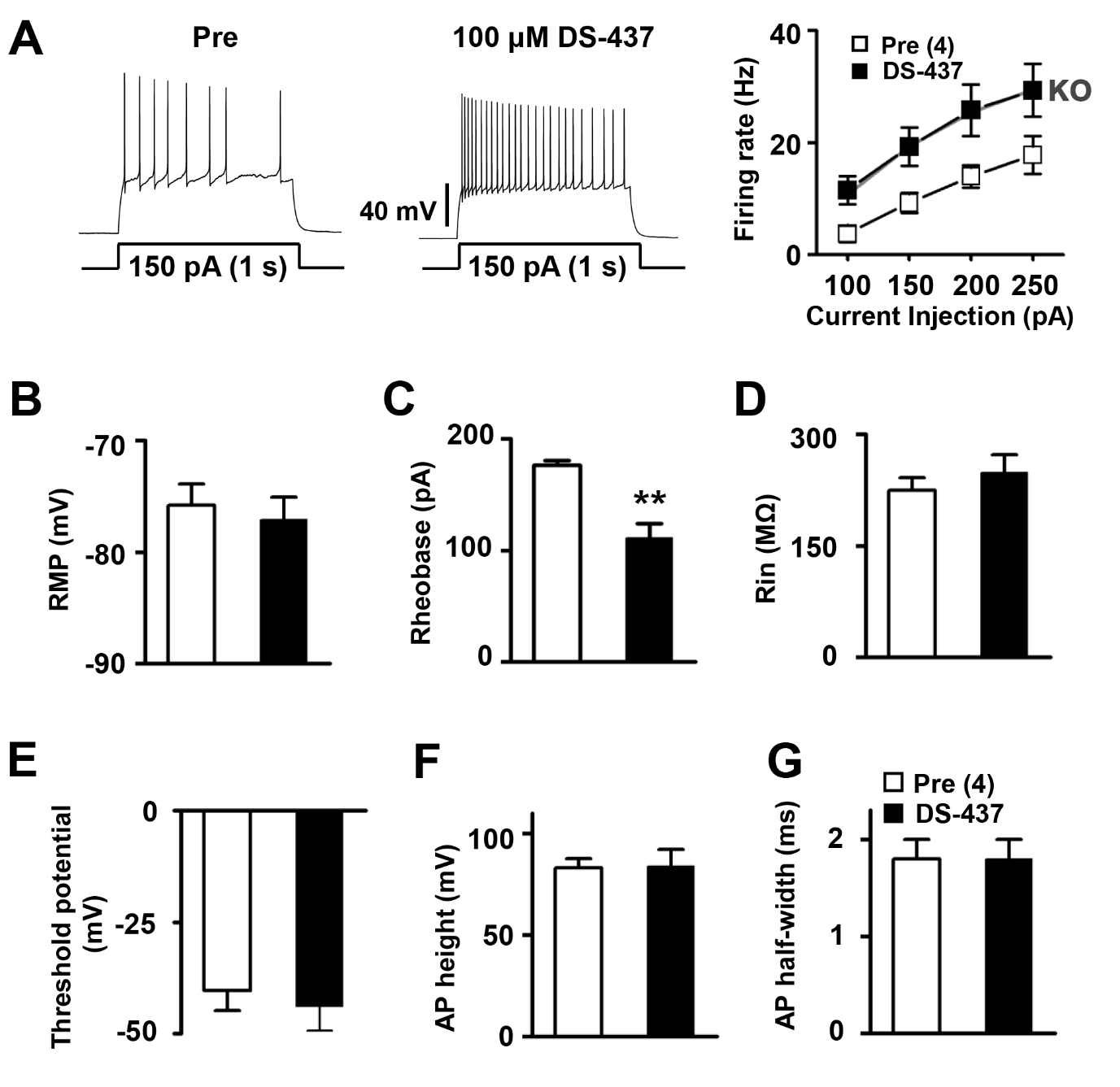
 Fig. S1**

**
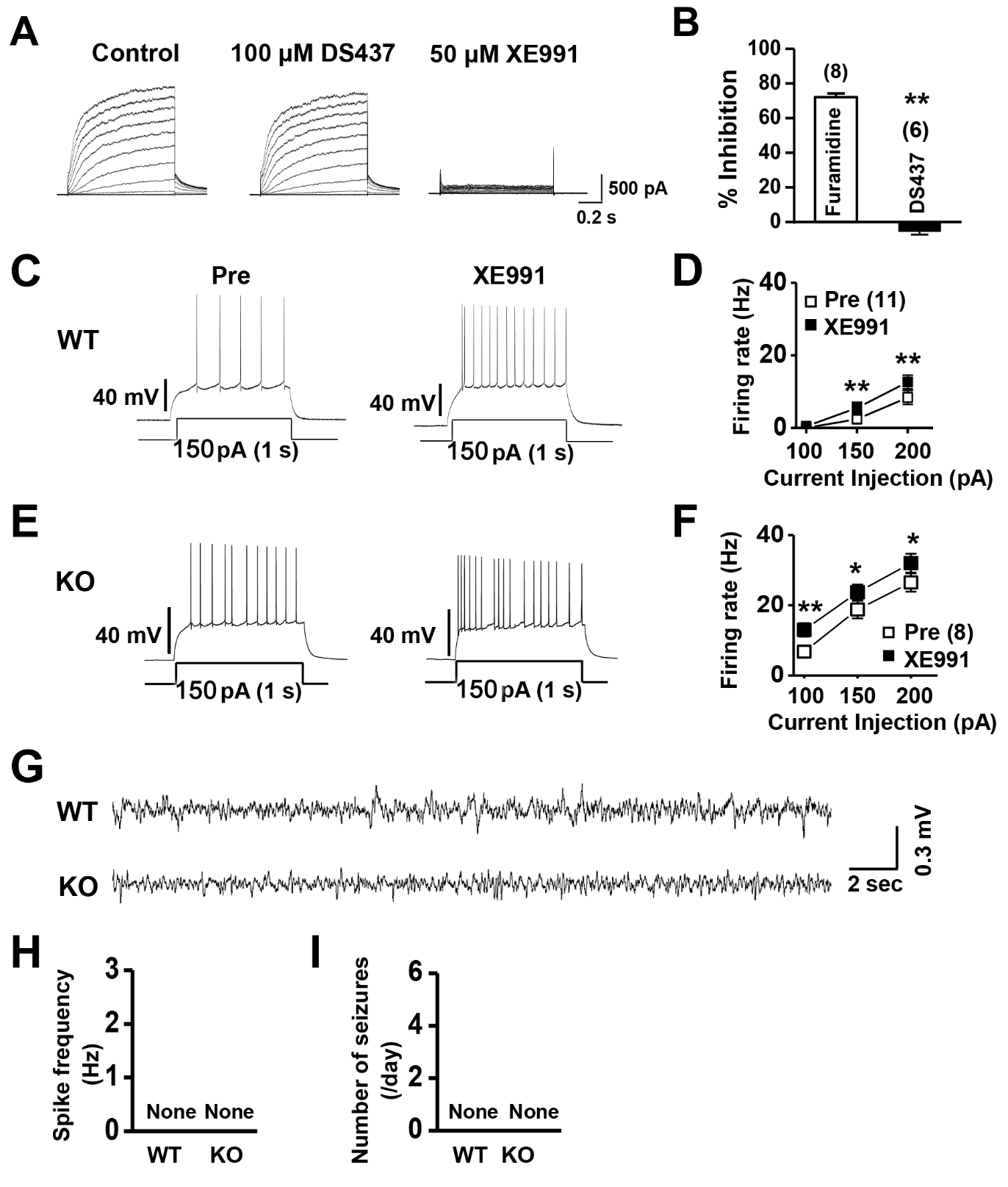
Fig. S2**

**

Fig. S3**

**Fig. S4**

**
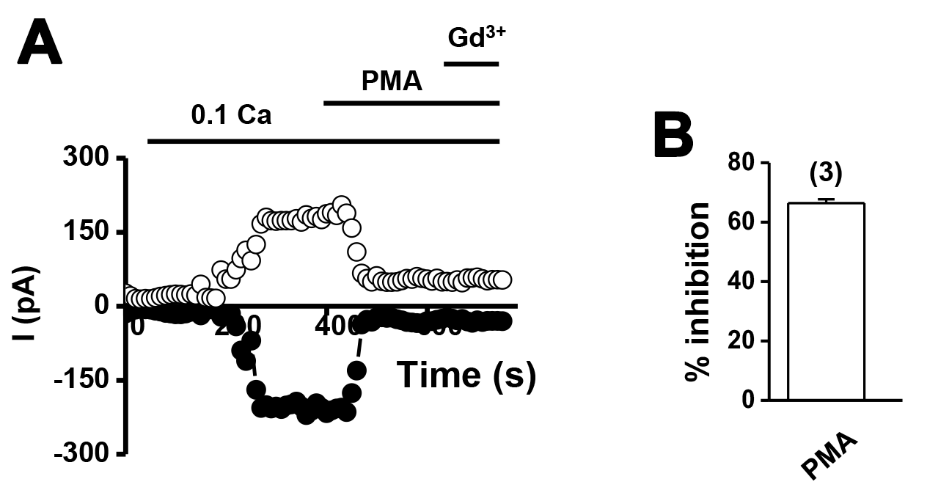
**

**Fig. S5**

**
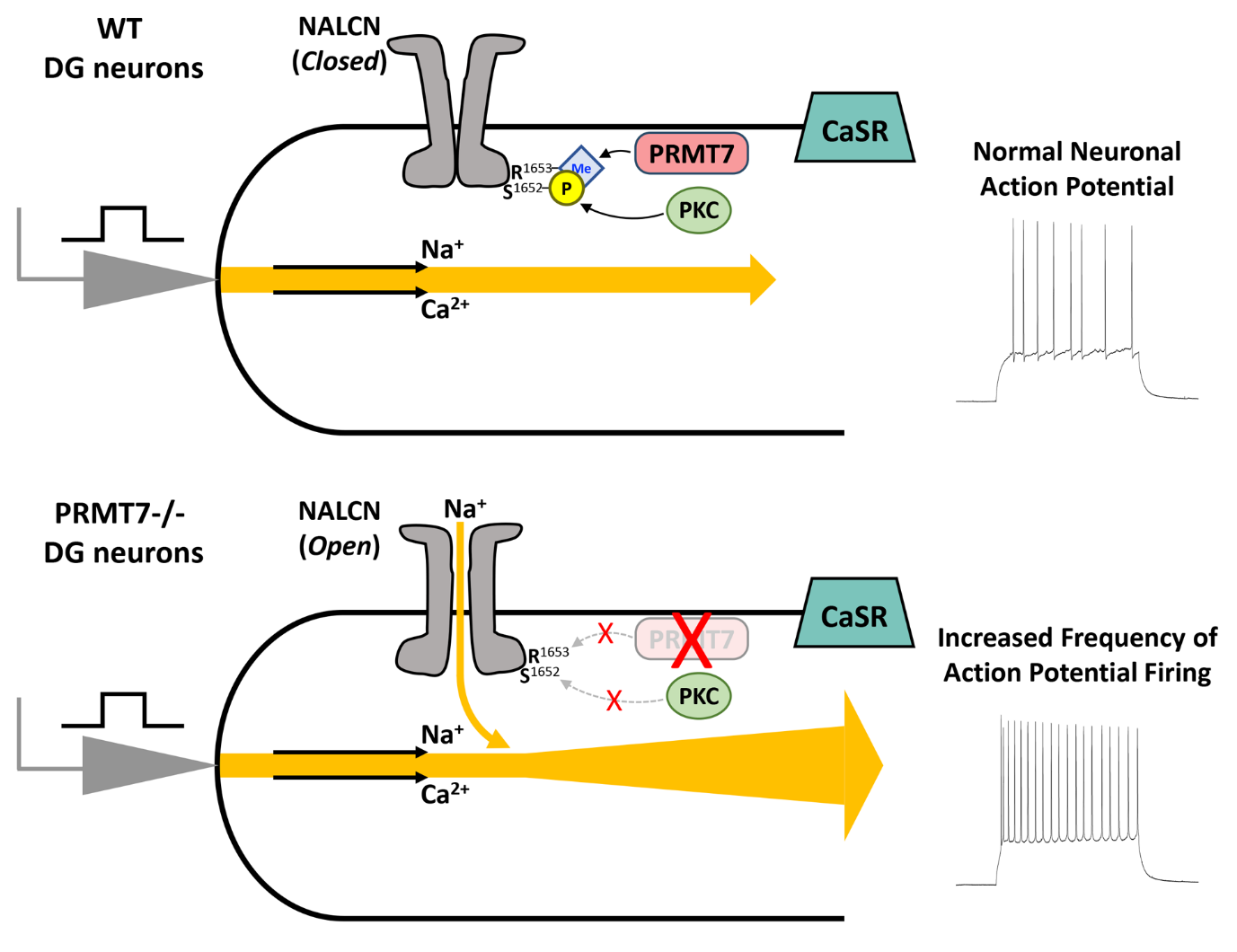
**
